# Clinical Outcomes of Mortality, Readmissions, and Ischemic Stroke Among Medicare Patients Undergoing Left Atrial Appendage Closure via Implanted Device

**DOI:** 10.1001/jamanetworkopen.2019.14268

**Published:** 2019-10-30

**Authors:** Rajesh Kabra, Saket Girotra, Mary Vaughan Sarrazin

**Affiliations:** 1 Division of Cardiology, Department of Internal Medicine, University of Tennessee Health Science Center, Memphis; 2 Department of Internal Medicine, University of Iowa College of Medicine, Iowa City; 3 Center for Comprehensive Access and Delivery Research and Evaluation, Iowa City Veterans Affairs Health Care System, Iowa City, Iowa

## Abstract

**Importance:**

Left atrial appendage closure (LAAC) has emerged as a nonpharmacological alternative for stroke prevention in patients with nonvalvular atrial fibrillation. Contemporary data regarding the characteristics and outcomes of patients undergoing this procedure are limited.

**Objective:**

To determine the characteristics, postprocedural mortality rate, and rates of readmission and ischemic stroke among Medicare beneficiaries who underwent LAAC via implanted device.

**Design, Setting, and Participants:**

This population-based cohort study used Medicare inpatient claims from January 2015 to December 2017 for 13 627 patients aged older than 65 years with a diagnosis of atrial fibrillation who underwent LAAC with an implanted device from January 2015 to November 2017. Data analyses were conducted from January through August 2019.

**Exposure:**

Left atrial appendage closure.

**Main Outcomes and Measures:**

Mortality rates during the index admission and for 1 year after LAAC were assessed. The 30-day readmission rate and the primary reasons for readmission were also examined. In addition, the likelihood of hospitalization for ischemic stroke within 180 days among 9231 patients for whom 6 months of follow-up data were available was evaluated.

**Results:**

Among the 13 627 patients undergoing LAAC, the mean (SD) age was 78.0 (6.3) years, 9406 (69.0%) were older than 75 years, 11 980 (87.9%) were white, and 5630 (41.3%) were women. The mean (SD) CHA_2_DS_2-_VASc score was 4.3 (1.4). Twenty-eight patients (0.2%) died during the index admission. Mortality rates were 0.6% (80 patients) at 30 days, 1.9% (262 patients) at 90 days, 4.0% (547 patients) at 180 days, and 7.5% (1027 patients) at 365 days. The 30-day readmission rate among 13 599 patients discharged alive was 9.4% (1284 patients). Among 9231 patients with 6 months of follow-up data, 111 (1.2%) experienced readmission for ischemic stroke or transient ischemic attack within 180 days of discharge.

**Conclusions and Relevance:**

In this cohort study of patients older than 65 years with atrial fibrillation who underwent LAAC with an implanted device, readmission rates for ischemic strokes over the course of 6 months were lower than expected given the patients’ CHA_2_DS_2-_VASc scores in the absence of anticoagulation therapy. Mortality at 1 year was higher than that reported in previous randomized clinical trials, likely because the patients in the present study were older, had a higher number of comorbidities, and were ineligible to receive warfarin.

## Key Points

**Question:**

What are the clinical outcomes among Medicare patients with atrial fibrillation undergoing left atrial appendage closure with an implanted device?

**Findings:**

In this cohort study of 13 627 patients with atrial fibrillation who underwent left atrial appendage closure, the rate of hospitalization for stroke at 6 months (1.2%) was significantly lower than expected according to their CHA_2_DS_2-_VASc scores in the absence of anticoagulation therapy. The 1-year mortality rate (7.5%) was higher than that in previous randomized clinical trials, likely because of the inclusion of older patients with more comorbidities who were ineligible to receive warfarin.

**Meaning:**

Left atrial appendage closure via an implanted device was associated with a decreased risk of stroke in patients with atrial fibrillation.

## Teaser

This cohort study of patients with atrial fibrillation who underwent left atrial appendage closure with an implanted device evaluates postprocedural mortality rates and rates of readmission and ischemic stroke.

## Introduction

Atrial fibrillation (AF) has emerged as a cardiovascular epidemic, with prevalence estimated to approach 12.1 million individuals in the United States by 2030.^1^ It increases the risk for stroke by 4- to 5-fold and is an independent risk factor for ischemic stroke severity, recurrence, stroke-related disability, and mortality. Anticoagulation therapy is the cornerstone for stroke prevention in patients with AF, according to their CHA_2_DS_2-_VASc (congestive heart failure; hypertension; ages ≥74 years [2 points]; diabetes; stroke, transient ischemic attack [TIA], or systemic embolism [2 points]; vascular disease; ages 65-74 years; sex [female]) risk scores.^2^ Left atrial appendage closure (LAAC) devices have emerged as nonpharmacological alternatives for stroke prevention in patients with AF.^3^ The Watchman device (Boston Scientific) was the first LAAC device that was compared with warfarin in 2 randomized clinical trials: PROTECT AF (Watchman Left Atrial Appendage System for Embolic Protection in Patients With Atrial Fibrillation; ClinicalTrials.gov identifier: NCT00129545)^4^ and PREVAIL (Evaluation of the WATCHMAN LAA Closure Device in Patients With Atrial Fibrillation vs Long Term Warfarin Therapy; ClinicalTrials.gov identifier: NCT01182441).^5^ The device received US Food and Drug Administration approval in 2015 for stroke prevention in patients with nonvalvular AF who are at high risk of stroke and are deemed suitable for short-term oral anticoagulation therapy but have appropriate rationale to seek nonpharmacologic alternatives to warfarin. To date, the real-world data outside clinical trials regarding the use and outcomes of LAAC devices are limited. To address this gap in knowledge, in this study, we describe the characteristics of patients undergoing LAAC via implanted device and examine the postprocedural outcomes of mortality, hospital readmissions, and ischemic stroke using Medicare data.

## Methods

### Study Population

The study was approved by the University of Iowa institutional review board, and the need for consent was waived because of the use of encrypted patient identifiers. This study follows the Strengthening the Reporting of Observational Studies in Epidemiology (STROBE) reporting guideline.^6^

The study used Centers for Medicare & Medicaid Beneficiary Summary (enrollment) and Medicare Provider and Analysis Review (inpatient claims) files from January 2015 through December 2017. Patients aged 66 years and older who underwent LAAC with the Watchman device during January 2015 through November 2017 were identified in hospital discharge records in the Medicare Provider and Analysis Review files according to *International Statistical Classification of Diseases and Related Health Problems, Tenth Revision *(*ICD-10*) procedure code 02L73DK (occlusion of left atrial appendage with intraluminal device, percutaneous approach). Currently, Watchman is the only intraluminal LAAC device approved by the US Food and Drug Administration for this procedure code. We obtained patient dates of death from the Beneficiary Summary File, with mortality information available up to 1 year after the LAAC. We only included patients older than 65 years who had at least 1 year of Medicare enrollment to identify comorbidities from the inpatient claims preceding LAAC.

Patient demographic characteristics (age, sex, and race/ethnicity) were identified on the Beneficiary Summary File. Comorbid conditions included in the CHA_2_DS_2-_VASc score were identified using an established algorithm according to *International Classification of Diseases, Ninth Revision, Clinical Modification *(*ICD-9-CM*) and *ICD-10* diagnoses on the LAAC hospital discharge record, as well as inpatient claims during the year preceding the LAAC admission.^7,8,9^

### Primary and Secondary Outcomes

The primary outcome was mortality during admission and within 1 year of LAAC. The secondary outcomes were readmissions within 30 days after LAAC discharge and reasons for readmission among patients discharged alive. To assess reasons for readmission, all first readmissions within 30 days of discharge were categorized according to the primary diagnosis code using the US Agency for Healthcare Research and Quality Clinical Classification System,^9^ which collapses individual *ICD-9-CM* and *ICD-10* codes into approximately 200 meaningful clinical categories. In addition, we evaluated the likelihood of admission for ischemic stroke or TIA within 180 days and the rate of admission for ischemic stroke or TIA per patient-year among patients discharged alive for whom at least 6 months of follow-up data were available. Admissions for ischemic stroke were identified by primary diagnosis on inpatient claims using *ICD-10* codes starting with I63, I65, and I66 for ischemic stroke and G45 for TIA.

### Statistical Analysis

Data analyses were conducted from January through August 2019. Summary statistics for patient characteristics were described as proportions for categorical variables (sex, race/ethnicity, and patient comorbidities), or using mean and SD for continuous variables (patient age and CHA_2_DS_2-_VASc score). Mortality was expressed as the percentage of patients who died within 30, 90, and 180 days. Readmission rates for ischemic stroke and TIA were reported as the percentage of patients admitted for stroke or TIA within 180 days (among patients discharged alive after LAAC), as well as the rate of stroke or TIA admissions per patient-year of follow-up. In the latter estimate, patients were censored because of death or end of the observation period (December 31, 2017). Confidence intervals around the rate of stroke admissions were generated assuming a Poisson distribution. Stroke admission rates were reported for a composite representing ischemic stroke or TIA, as well as separately for TIA and ischemic stroke. Finally, we assessed trends in mortality and readmission rates over time and by CHA_2_DS_2_-VASc score using the Cochran-Armitage test for trend. Statistical analyses were performed using SAS statistical software version 9.4 (SAS Institute). All statistical testing was 2-tailed, with *P* < .05 designating statistical significance.

## Results

Overall, 13 627 patients with AF underwent LAAC with Watchman implantation from January 2015 through November 2017. The mean (SD) age was 78.0 (6.3) years, 7997 (59.7%) were men, 5630 (41.3%) were women, 9406 (69.0%) were older than 75 years, and their mean (SD) CHA_2_DS_2_-VASc score was 4.3 (1.4), which would yield an expected annual ischemic stroke risk of 4.8%, according to previous work.^10^ There were 11 980 white patients (87.9%), 455 black patients (3.3%), and 523 Hispanic patients (3.8%). Table 1 compares the patients undergoing LAAC in our study with those in the randomized clinical trials.^4,5^ Compared with the randomized trials, the patients in the current study were older (mean [SD] age, 78.0 [6.3] years vs 71.7 [8.8] years and 74.0 [7.4] years); with a higher percentage of women; higher prevalence of congestive heart failure (41.9% vs 26.8% and 23.4%), hypertension (91.8% vs 89.6% and 88.5%), and diabetes (37.1% vs 24.4% and 33.8%); and a higher mean (SD) CHA_2_DS_2_-VASc score (4.4 [1.4] vs 3.4 [1.5] and 3.8 [1.2]).

**Table 1.** Demographic Characteristics of Patients Undergoing Left Atrial Appendage Closure With Watchman Device in the Present Study Compared With the PROTECT AF and PREVAIL Randomized Clinical Trials

Characteristic	Present Study (N = 13 627), Patients, No. (%)	Previous Trials, Patients, %	
		PROTECT AF^4^ (N = 463)	PREVAIL^5^ (N = 269)
Age, mean (SD), y	78.0 (6.3)	71.7 (8.8)	74 (7.4)
Male	7997 (59.7)	70.4	67.7
CHA_2_DS_2_-VASc score, mean (SD)	4.4 (1.4)	3.4 (1.5)	3.8 (1.2)
Race/ethnicity			
White	11 980 (87.9)	91.8	94.1
Black	455 (3.3)	1.3	2.2
Hispanic	523 (3.8)	5.4	2.2
Other	669 (4.9)	1.5	1.1
Congestive heart failure	5708 (41.9)	26.8	23.4
Hypertension	12 507 (91.8)	89.6	88.5
Age ≥75 y	9406 (69.0)	36.9	46.5
Diabetes	5055 (37.1)	24.4	33.8
Prior stroke	2027 (14.9)	20.1	29.7

Abbreviations: CHA_2_DS_2_-VASc, congestive heart failure; hypertension; ages ≥74 years (2 points); diabetes; stroke, transient ischemic attack, or systemic embolism (2 points); vascular disease; ages 65-74 years; sex (female); PREVAIL, Evaluation of the WATCHMAN LAA Closure Device in Patients With Atrial Fibrillation vs Long Term Warfarin Therapy; PROTECT AF, Watchman Left Atrial Appendage System for Embolic Protection in Patients With Atrial Fibrillation.

During the hospital admission for LAAC, 28 patients (0.2%) died. Over the observation period, mortality rates were 0.6% (80 patients) at 30 days, 1.9% (262 patients) at 90 days, 4.0% (547 patients) at 180 days, and 7.5% (1027 patients) at 1 year (Table 2). The 30-day readmission rate among 13 599 patients discharged alive was 9.4% (1284 patients). Among 9231 patients with 6 months of follow-up data, 111 (1.2%) experienced readmission for ischemic stroke or TIA within 180 days of discharge, with 84 patients (0.9%) readmitted for ischemic stroke and 29 patients (0.3%) readmitted for TIA. The corresponding admission rates were 2.5 readmissions per 100 patient-years (95% CI, 2.2-2.8 readmissions per 100 patient-years) for stroke or TIA, 1.9 readmissions per 100 patient-years (95% CI, 1.7-2.2 readmissions per 100 patient-years) for ischemic stroke only, and 0.6 readmissions per 100 patient-years (95% CI, 0.5-0.8 readmissions per 100 patient-years) for TIA only.

**Table 2.** Mortality After Left Atrial Appendage Closure and Admissions for Ischemic Stroke, Overall and by CHA_2_DS_2_-VASc Score

Parameter	Mortality					Ischemic Stroke Within 180 d, No. (%)			
	Patients, No.	No. (%)				Patients, No.	No. (%)		
		Within 30 d	Within 90 d	Within 180 d	Within 365 d		Ischemic Stroke or TIA	Ischemic Stroke	TIA
Overall	13 627	80 (0.6)	262 (1.9)	547 (4.0)	1027 (7.5)	9231	111 (1.2)	84 (0.9)	29 (0.3)
By CHA_2_DS_2_-VASC score									
0-3	3608	13 (0.4)	38 (1.1)	81 (2.3)	135 (4.0)	545	13 (0.85)	8 (0.3)	1 (0.2)
4-5	7495	44 (0.6)	137 (1.8)	287 (3.8)	577 (7.7)	4702	65 (1.4)	52 (1.0)	16 (0.3)
≥6	2524	23 (0.9)	87 (3.5)	179 (7.1)	304 (12.0)	3984	30 (1.8)	24 (1.5)	1 (0.5)
*P* value		.006	<.001	<.001	<.001		<.001	<.001	.10

Abbreviations: CHA_2_DS_2_-VASc, congestive heart failure; hypertension; ages ≥74 years (2 points); diabetes; stroke, transient ischemic attack, or systemic embolism (2 points); vascular disease; ages 65-74 years; sex (female); TIA, transient ischemic attack.

The mortality and stroke readmission rates in the present study compared with those of previous studies^4,5,11,12,13,14^ are shown in Table 3. The previous studies had ischemic stroke rates of 1.6%, 1.7%, and 1.1% and mortality rates of 3.6%, 4.6%, and 9.8%, respectively. We discuss these comparisons in greater detail later in the article.

**Table 3.** Comparison of Outcomes of Ischemic Stroke and Mortality After Left Atrial Appendage Closure With Implanted Device With Prior Studies

Outcome	Rate/100 Patient-Years, %			
	Present Study (N = 13 627)	PROTECT AF^4^ and PREVAIL^5^ (N = 732)	ASAP^12,13^ (N = 150)	EWOLUTION^14^ (N = 1025)
Ischemic stroke	1.2	1.6	1.8	1.1
Mortality at 1 y	7.5	3.6	4.6	9.8

Abbreviations: ASAP, ASA Plavix Feasibility Study With Watchman Left Atrial Appendage Closure Technology; EWOLUTION, Registry on Watchman Outcomes in Real Life Utilization; PREVAIL, Evaluation of the WATCHMAN LAA Closure Device in Patients With Atrial Fibrillation vs Long Term Warfarin Therapy; PROTECT AF, Watchman Left Atrial Appendage System for Embolic Protection in Patients With Atrial Fibrillation.

We further examined mortality and readmission rates for stroke and TIA by CHA_2_DS_2_-VASc score (Table 2 and Figure). All outcomes were more frequent among patients with higher CHA_2_DS_2_-VASc scores. In particular, among those with higher CHA_2_DS_2_-VASc scores, mortality rates were higher within 90 days (score 0-3, 1.1%; score 4-5, 1.8%, score ≥6, 3.5%; *P* < .001), within 180 days (score 0-3, 2.3%; score 4-5, 3.8%; score ≥6, 7.1%; *P* < .001), and within 365 days (score 0-3, 4.0%; score 4-5, 7.7%; score ≥6, 12.0%; *P* < .001). We compared the observed rates of ischemic stroke after LAAC via implanted device in our study with expected stroke rates in the absence of anticoagulation therapy according to CHA_2_DS_2_-VASc scores, using data from prior studies.^10^ We noted a 60% to 69% relative reduction in stroke risk after LAAC, compared with the risk in the absence of anticoagulation therapy (Figure).

**Figure.**
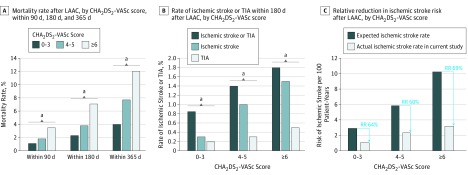
Mortality, Rate of Ischemic Stroke and Transient Ischemic Attack (TIA), and Relative Reduction in Ischemic Stroke Risk After Left Atrial Appendage Closure (LAAC) According to CHA_2_DS_2_-VASc Score A, Mortality rates within 90, 180, and 365 days after LAAC are shown according to CHA_2_DS_2_-VASc (congestive heart failure; hypertension; ages ≥74 years [2 points]; diabetes; stroke, transient ischemic attack, or systemic embolism [2 points]; vascular disease; ages 65-74 years; sex [female]) score. B, Rate of ischemic stroke or TIA within 180 days after LAAC according to the CHA_2_DS_2_-VASc score is shown. C, Relative reduction (RR) in ischemic stroke risk after LAAC according to the CHA_2_DS_2_-VASc score is shown. For CHA_2_DS_2_-VASc score of 0 to 3, the expected ischemic stroke rate was 2.90 and the actual rate was 1.05 per 100 patient-years; for CHA_2_DS_2_-VASc scores of 4 to 5, the expected ischemic stroke rate was 5.84 and the actual rate was 2.31 per 100 patient-years; and for CHA_2_DS_2_-VASc scores of 6 or more, the expected ischemic stroke rate was 10.24 and the actual rate was 3.18 per 100 patient-years. ^a^Statistically significant difference (*P* < .05).

The most frequent primary diagnoses for readmissions within 30 days were gastrointestinal hemorrhage (134 patients [1.0%]), hypertension with complications (111 patients [0.8%]), cardiac dysrhythmias (103 patients [0.8%]), complications from a device or surgical procedure (84 patients [0.6%]), and septicemia (68 patients [0.5%]). There were 28 readmissions (0.30%) for hemorrhagic stroke within 180 days after LAAC via implanted device.

## Discussion

To our knowledge, this is the largest study outside clinical trial to assess the characteristics of patients with AF undergoing LAACs and the associated outcomes of mortality, readmissions, and stroke. We noted that, compared with previous randomized clinical trials,^4,5^ our study had a higher percentage of women, and the patients had higher numbers of comorbidities and higher CHA_2_DS_2_-VASc scores. The risk of readmission for stroke (1.2% at 6 months) was significantly lower than that expected according to the mean (SD) CHA_2_DS_2-_VASc score of 4.3 (1.4) in the absence of anticoagulation therapy, which would yield an expected annual ischemic stroke risk of 4.8%, according to previous work.^10^

A recent meta-analysis^11^ of the PROTECT AF^4^ and PREVAIL^5^ randomized clinical trials compared 5-year outcomes for the 732 patients who underwent LAAC with the Watchman device with 382 patients in the control group treated with warfarin. In the LAAC groups of those studies,^4,5^ the incidence of ischemic stroke or systemic embolism was 1.6% per 100 patient-years, and the overall mortality rate was 3.6% per 100 patient-years. Left atrial appendage closure was associated with lower risk of all-cause mortality, as well as cardiovascular or unknown death, compared with warfarin. In the present study, readmission for ischemic stroke occurred in 111 of 9231 patients (1.2%) within 180 days after LAAC implantation. We observed a mortality rate of 7.5% at 1 year, and 28 patients (0.2%) died during the index admission for LAAC. There can be several explanations for the slightly higher rates of stroke and mortality in our study compared with the randomized clinical trials.^4,5^ Our study included an older patient population (aged >65 years) with higher numbers of comorbidities and higher CHA_2_DS_2_-VASc scores. Patients in the randomized clinical trials had to be eligible to receive warfarin to participate, which would likely preselect a healthier population. In contrast, eligibility for long-term anticoagulation therapy is not a requirement for the device implantation in clinical practice.

In the ASAP (ASA Plavix Feasibility Study With Watchman Left Atrial Appendage Closure Technology) nonrandomized study,^12,13^ 150 patients with AF who were not eligible for oral anticoagulation therapy underwent LAAC. Patients received aspirin and clopidogrel for 6 months after implantation and aspirin thereafter. The annualized ischemic stroke and systemic embolization rate was 1.8%, and the mortality rate at 1 year was 4.6%. Another long-term nonrandomized prospective real-world study from Europe, EWOLUTION (Registry on Watchman Outcomes in Real Life Utilization),^14^ reported 1-year outcome data for 1025 patients undergoing LAAC. The annual rate of ischemic stroke was 1.1% (15 of 1325 patient-years), and the 1-year mortality rate was 9.8%. Many of these patients were not eligible for anticoagulation therapy and underwent LAAC as a last resort option for stroke prevention. In both studies,^12,13,14^ the rate of stroke was lower than that in the present study. However, as observed in the present study, the 1-year mortality rate was significantly higher than those in the randomized studies,^4,5^ possibly because of the inclusion of patients with higher numbers of comorbidities.

### Limitations

This study is retrospective and based on Medicare claims data, which have inherent limitations. We only included patients aged older than 65 years; therefore, our results cannot be extrapolated to younger patient populations. Specific details regarding LAAC via device implantation, immediate complications, preimplantation and postimplantation antithrombotic therapy, or postimplantation left atrial appendage imaging results were not available. The exact cause of death and whether it was related to the Watchman implantation procedure were unclear.

## Conclusions

In this retrospective real-world study of Medicare patients aged older than 65 years with AF, LAAC via implanted device was associated with a decreased risk of admission for stroke compared with the expected risk without anticoagulation therapy. The risks of 1-year mortality and stroke at 6 months were higher than those in previous randomized clinical trials, likely because the population in the current study was older and had more comorbidities and higher CHA_2_DS_2_-VASc scores. Although LAAC provides an attractive nonpharmacological option for stroke prevention in patients with AF who are not candidates for long-term anticoagulation therapy, further prospective long-term real-world studies are needed to monitor its safety and efficacy.
