# Errors in Results, Discussion, and Table 3

**DOI:** 10.1001/jamanetworkopen.2019.17818

**Published:** 2019-11-22

In the Original Investigation titled “Clinical Outcomes of Mortality, Readmissions, and Ischemic Stroke Among Medicare Patients Undergoing Left Atrial Appendage Closure via Implanted Device,”^1^ published October 30, 2019, there were errors in the third paragraph of the Results, the third paragraph of the Discussion, and Table 3. In one of the previous trials mentioned in the Results and Discussion, the rate of ischemic stroke and systemic embolization was 1.7%, not 1.8%, and the mortality rate at 1 year was 5.0%, not 4.6%; these errors also appeared in Table 3. In addition, the ischemic stroke rate of the study by Kabra et al shown in Table 3 should have been 1.9%, not 1.2%. This article has been corrected.^1^
